# High-mobility junction field-effect transistor via graphene/MoS_2_ heterointerface

**DOI:** 10.1038/s41598-020-70038-6

**Published:** 2020-08-04

**Authors:** Taesoo Kim, Sidi Fan, Sanghyub Lee, Min-Kyu Joo, Young Hee Lee

**Affiliations:** 1 Center for Integrated Nanostructure Physics (CINAP), Institute for Basic Science (IBS), Sungkyunkwan University, Suwon, 16419 Republic of Korea; 2 Department of Energy Science, Department of Physics, Sungkyunkwan University, Suwon, 16419 Republic of Korea; 3 Department of Applied Physics, Sookmyung Women’s University, Seoul, 04310 Republic of Korea; 4 Institute of Advanced Materials and Systems, Sookmyung Women’s University, Seoul, 04310 Republic of Korea

**Keywords:** Nanoscience and technology, Physics

## Abstract

Monolayer molybdenum disulfide (MoS_2_) possesses a desirable direct bandgap with moderate carrier mobility, whereas graphene (Gr) exhibits a zero bandgap and excellent carrier mobility. Numerous approaches have been suggested for concomitantly realizing high on/off current ratio and high carrier mobility in field-effect transistors, but little is known to date about the effect of two-dimensional layered materials. Herein, we propose a Gr/MoS_2_ heterojunction platform, i.e., junction field-effect transistor (JFET), that enhances the carrier mobility by a factor of ~ 10 (~ 100 cm^2^ V^−1^ s^−1^) compared to that of monolayer MoS_2_, while retaining a high on/off current ratio of ~ 10^8^ at room temperature. The Fermi level of Gr can be tuned by the wide back-gate bias (*V*_BG_) to modulate the effective Schottky barrier height (SBH) at the Gr/MoS_2_ heterointerface from 528 meV (*n*-MoS_2_/*p*-Gr) to 116 meV (*n*-MoS_2_/*n*-Gr), consequently enhancing the carrier mobility. The double humps in the transconductance derivative profile clearly reveal the carrier transport mechanism of Gr/MoS_2_, where the barrier height is controlled by electrostatic doping.

## Introduction

Graphene (Gr), which consists of carbon atoms in a planar two-dimensional (2D) array, provides a platform for a new era of 2D electronics to replace mainstream silicon-driven semiconductors owing to its excellent mobility of up to 200,000 cm^2^ V^−1^ s^−1^ at room temperature^1,2^. Yet, the poor switching due to the zero bandgap of Gr remains a critical issue hindering practical applications, despite extensive efforts such as functionalization^3–5^, chemical doping^6^^,^ graphene nanoribbon formation^7–10^, and the development of bilayer graphene with a dual-gate structure^11–13^. Hexagonal molybdenum disulfide (MoS_2_) comprising one molybdenum atom with two surrounding sulfur atoms exhibits *n*-type semiconducting behavior with a high on/off current ratio exceeding 10^8^ [ref. ^14,16^]. Nevertheless, the moderate carrier mobility (0.1 to 10 cm^2^ V^−1^ s^−1^)^14,17–19^, is several orders of magnitude lower than that of Gr, thereby limiting the potential of MoS_2_ for high-speed switching device applications.

Thus far, numerous approaches have been introduced to enhance electrical performance of MoS_2_ based device, for example, gate dielectric and contact resistance engineering have been suggested for enhancing the carrier mobility of MoS_2_ to achieve high switching performance. High-κ (*e.g.*, Al_2_O_3_, HfO_2_) dielectrics suppress the surface reaction and enhance the dielectric screening effect^20–22^, thereby enhancing the carrier mobility of MoS_2_ (reaching 81 cm^2^ V^−1^ s^−1^)^23^. Sub-stoichiometric high-κ dielectrics generate more carriers in the thin MoS_2_ layer, effectively screening out various Coulomb scattering sources derived from polymer residues, charged impurities, and interface states^15,24^. Encapsulating *h*-BN is a prospectively ideal approach^25^^,^ but mechanical exfoliation is impractical because of the scalability of the cleaved film. Contact resistance engineering by utilizing a low work-function metal such as titanium^26^ or scandium^27^ is one alternative approach for achieving high carrier mobility. Despite lowering the Schottky barrier height and improving the carrier injection of multilayer MoS_2_ significantly, the choice of contact metals for the monolayer in this approach is very limited due to Fermi level pinning depending on the surface states or defect sites of the metal/MoS_2_ interface. Recently, it has been successfully demonstrated that monolayer Gr enables to suppress the Schottky barrier height sufficiently at Gr/MoS_2_ heterointerface as an ideal contact material for 2D electronic materials^28^, which facilitates diverse 2D heterostructures^29^. Nevertheless, the limited carrier mobility of 2D materials still constrains their practical electronic applications, requiring different approaches.

We propose an ideal device platform based on a junction field-effect transistor (JFET) architecture featuring a Gr/MoS_2_ heterointerface, where the carrier mobility of MoS_2_ is enhanced by a factor of 10, while maintaining a high on/off current ratio of up to 10^8^ at room temperature. The Schottky barrier height (SBH) governs the carrier injection and carrier mobility in the Gr/MoS_2_ heterojunction device. The low SBH regime at *n*-Gr/*n*-MoS_2_ provides an additional Gr conduction path for MoS_2_, leading to *μ*_FE_ ~ 100 cm^2^ V^−1^ s^−1^, whereas the high SBH regime at *p*-Gr/*n*-MoS_2_ blocks the contribution of Gr to MoS_2_, leading to *μ*_FE_ ~ 10 cm^2^ V^−1^ s^−1^, similar to that of the pure MoS_2_-based device.

## Results and discussions

**Figure 1:**
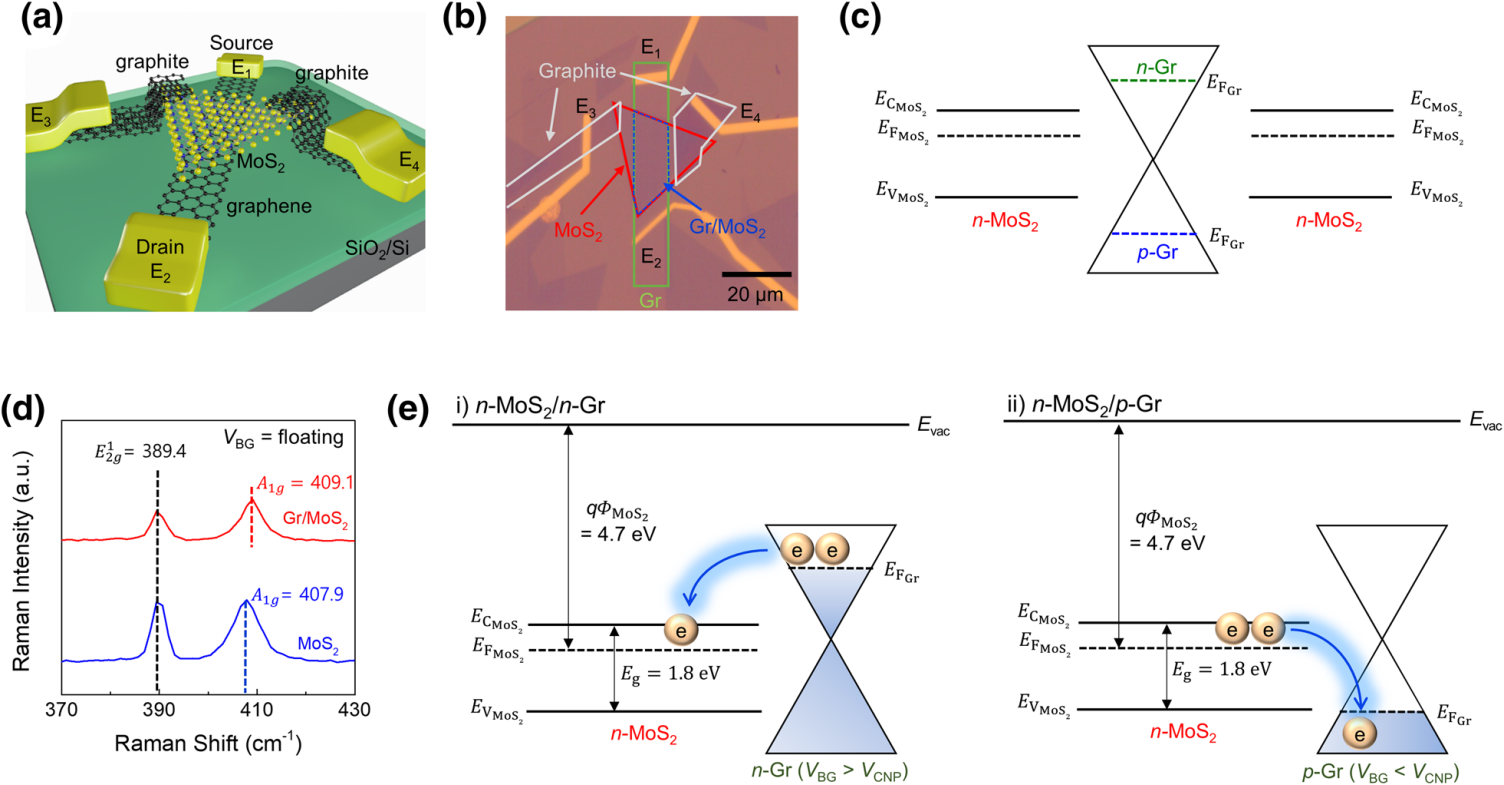
Device structure and Raman characterization of the Gr/MoS_2_ heterostructure. (**a**) Schematic of a two-terminal device with the Gr/MoS_2_ heterostructure with graphite as the electrical contact. (**b**) Optical microscopic image of the heterostructure device. Electrode E_1_ and E_2_ are the source/drain contacts for the bottom Gr (green rectangular) layer, and E_3_ and E_4_ are electrically connected to the upper MoS_2_ layer via the graphite electrode. (**c**) Simplified band diagram of the Gr/MoS_2_ heterostructure in the lateral direction with graphite contacts (E_3_ and E_4_). (**d**) Optical Raman spectra of MoS_2_ and the Gr/MoS_2_ heterostructure. Blue-shift of A_1g_ peak indicates charge transfer from MoS_2_ to Gr. **(e)** Band diagram of charge transfer direction at the Gr/MoS_2_ heterointerface under different gate bias conditions. ${E}_{vac}$, ${q\Phi }_{{MoS}_{2}}$, ${E}_{{C}_{{MoS}_{2}}}$, ${E}_{{F}_{{MoS}_{2}}}$, ${E}_{{V}_{{MoS}_{2}}}$, ${E}_{g}$, and ${E}_{{F}_{Gr}}$ indicate the vacuum level, work function of MoS_2_, conduction band of MoS_2_, Fermi level of MoS_2_, valence band of MoS_2_, band-gap, and Fermi level of Gr, respectively.

To simultaneously achieve high carrier mobility and on/off current ratio, chemical vapor deposited (CVD) monolayer MoS_2_ was intentionally stacked on top of a monolayer Gr strip that was also grown by CVD. Properly chosen mechanically exfoliated graphite flakes were employed as the source and drain contacts to protect the MoS_2_ channel during metal deposition, thereby circumventing the Fermi level pinning effect^30^. Fig. 1 presents a schematic of the conceptual Gr/MoS_2_ heterostructure device with the graphite contact (Fig. 1a), the corresponding optical image (Fig. 1b), and the simplified band diagram (Fig. 1c) in the lateral direction with graphite contacts (E_3_ and E_4_), respectively. The detailed device fabrication procedure is described in the Methods section.

The optical Raman spectrum of the Gr/MoS_2_ heterostructure (Fig. 1d) was acquired under argon atmosphere to prevent unintended oxidation or degradation. The ${E}_{2g}^{1}$ peaks (~ 389.4 cm^−1^) of bare MoS_2_ and the Gr/MoS_2_ heterostructure are similarly positioned, implying negligible strain in the developed device. The blue-shift of the ${A}_{1g}$ peak for the heterostructure with respect to that of MoS_2_ indicates electron charge transfer from MoS_2_ to Gr, as reported previously^31,32^. The relevant band diagram depending on the carrier type of Gr (Fig. 1e) illustrates the vertical charge transfer direction based on the gate bias. In the *n*-Gr/*n*-MoS_2_ regime, the electron population in *n*-Gr is transferred to *n*-MoS_2_, leading to a negligible vertical SBH, whereas in the *p*-Gr/*n*-MoS_2_ regime, electrons in *n*-MoS_2_ are transferred to *p*-Gr, resulting in a large vertical SBH, as discussed later. This charge transfer model explains the key carrier transport mechanism in the developed heterostructure device associated with the vertical SBH at the Gr/MoS_2_ heterojunction.

**Figure 2:**
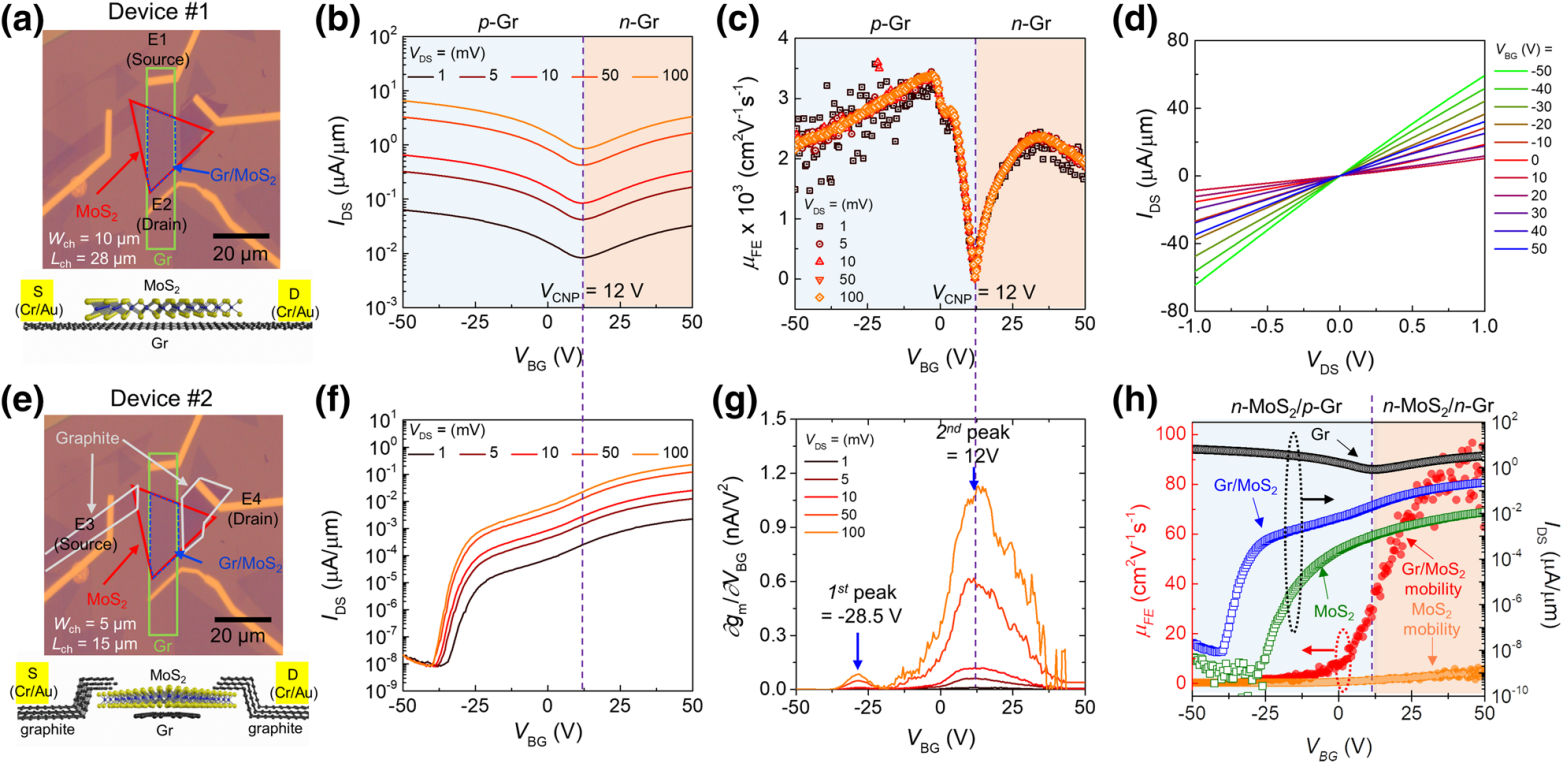
Electrical characteristics of Gr/MoS_2_ heterostructure device. (**a**‒**d**) Gr/MoS_2_ heterostructure device employing Gr contact with E_1_ (source) and E_2_ (drain) (Device #1: Gr-Gr/MoS_2_-Gr, *L*/*W* = 28 μm/10 μm). (**a**) Optical device image, *V*_DS_-dependent (**b**) *I*_DS_‒*V*_BG_ transfer curves (*V*_CNP_ = 12 V) and (**c**) *μ*_FE_, respectively. (**d**) *V*_BG_-dependent *I*_DS_‒*V*_DS_ output characteristic curves. (**e**‒**g**) Gr/MoS_2_ heterostructure device employing MoS_2_ contact with E_3_ and E_4_ (Device #2: graphite-MoS_2_-Gr/MoS_2_-MoS_2_-graphite, *L*/*W* = 15 μm/5 μm). (**e**) Optical device image, *V*_DS_-dependent (**f**) transfer curves and (**g**) corresponding derivative of *g*_m_ (= ∂*g*_m_/∂*V*_BG_) curves. The 1st and 2nd peak positions of *V*_BG_ indicate the *V*_FB_ of MoS_2_ and *V*_CNP_ of Gr. (**h**) Comparison of transfer curves of the three devices (Gr, MoS_2_, Device #2) (right *y*-axis) and the *μ*_FE_ (left *y*-axis) of MoS_2_ and Device #2 at *V*_DS_ = 0.1 V. An enhancement of *μ*_FE_ (~ 100 cm^2^ V^−1^ s^−1^) is clearly observed in the *n*-MoS_2_/*n*-Gr regime.

We first examined the back-gate bias (*V*_BG_)-dependent drain current (*I*_DS_) of graphene (Device #1: Gr-Gr/MoS_2_-Gr using metal electrodes E_1_ and E_2_) in the Gr/MoS_2_ heterostructure as displayed in Fig. 2a. The transfer curves based on the drain-source voltage (*V*_DS_) (Fig. 2b) demonstrate the ambipolar characteristics of the Gr in Device #1. The charge neutrality point (*V*_CNP_) is found at *V*_BG_ = 12 V regardless of *V*_DS_. This positive *V*_CNP_ indicates dominant electron transfer from Gr to the positive fixed oxide traps in the SiO_2_/Si substrate^31,33,34^. The field-effect mobility is described as *μ*_FE_ = *g*_m_ (*L*/*W*) *C*_OX_^−1^ *V*_DS_^−1^, where *g*_m_, *L*/*W*, and *C*_OX_ denote the transconductance (= ∂*I*_DS_/∂*V*_BG_), channel length-to-width ratio, and oxide capacitance per unit area, respectively. The maximum *μ*_FE_ is ~ 2,500 cm^2^ V^−1^ s^−1^ for *n*-Gr and ~ 3,500 cm^2^ V^−1^ s^−1^ for *p*-Gr (Fig. 2c). The *μ*_FE_ range is similar to that of the bare Gr device on SiO_2_^35^. The collapse of the *V*_DS_-dependent *μ*_FE_ clearly mirrors the negligible SBH effect. Further, super-linear *I*_DS_‒*V*_DS_ output characteristic curves were obtained because of the high tunability of the Fermi-level of Gr (Fig. 2d), confirming the low SBH or nearly ohmic contact.

Investigation of the Gr/MoS_2_ heterostructure device with the MoS_2_ contact in the two graphite electrodes (Device #2: graphite-MoS_2_-Gr/MoS_2_-MoS_2_-graphite using metal electrodes E_3_ and E_4_) (see Fig. 2e), shows a distinct current path difference comparing to Device #1. Because the conductivity of MoS_2_ is much lower than that of Gr or the Gr/MoS_2_ heterojunction in this case, the bare MoS_2_ dominates the carrier transport of Device #2, in contrast with Device #1. The *I*_DS_‒*V*_BG_ transfer curves (Fig. 2f) show clear switching behavior in the negative *V*_BG_ regime, originating primarily from the bare MoS_2_ component in Device #2. Notably, in the positive *V*_BG_ regime, a hump associated with a rapid enhancement of *I*_DS_ appears, independent of *V*_DS_. This strongly implies the presence of two different conduction mechanisms or conducting paths, similar to double-gate Si and junctionless Si transistors^36,37^.

To gain insight into the carrier transport mechanism of Device #2 in detail, the flat-band voltage (*V*_FB_) was determined from the second-derivative of the current (Fig. 2g)^38^. The first peak at *V*_BG_ =  − 28.5 V is attributed to the turn-on voltage of MoS_2_. The second peak at *V*_BG_ = 12 V is ascribed to the underlying Gr in Device #2, as confirmed by the identical *V*_CNP_ of Gr (Fig. 2c). The coincident position of the second hump for *V*_FB_ and *V*_CNP_ further rationalizes the presence of second conducting path Device #2. Since it has theoretically and experimentally been suggested that the vertical SBH of Gr/MoS_2_ can be modulated by electrostatic doping^39–42^, the bare MoS_2_ consequently governs the carrier transport of the device exclusively when *V*_BG_ ≤ *V*_CNP_ (*i.e.*, *p*-Gr/*n*-MoS_2_) at high vertical SBH. Another current path involving Gr in addition to MoS_2_ is provided at *V*_BG_ ≥ *V*_CNP_ (*i.e.*, *n*-Gr/*n*-MoS_2_) with low vertical SBH, leading to *μ*_FE_ for Device #2.

To have a better picture, *μ*_FE_ of heterostructure (Device #2) and bare MoS_2_ (Device #5 with metal electrodes E_9_ and E_10_, see Supplementary Note 1 and Fig. S1) at *V*_DS_ = 0.1 V is compared together in Fig. 2h. In principle, the current onset voltage for Device #2 and Device #5 should be identical because the bare MoS_2_ in both of them limits the carrier transport in the subthreshold regime. We ascribed this mismatched current onset voltage in Fig. 2h between the Gr/MoS_2_ (Device #2) and MoS_2_ (Device #5) to the different effective SBHs. While the MoS_2_ (Device #5) has a Cr/Au contact to MoS_2_, the MoS_2_/Gr (Device #2) possesses a graphite contact to MoS_2_, respectively. The maximum *μ*_FE_ of Device #5 ranges from 8 to 10 cm^2^ V^−1^ s^−1^ at *V*_BG_ = 50 V, which reaches to 16–20 cm^2^ V^−1^ s^−1^ after contact resistance correction (see Supplementary Fig. S2). Meanwhile, the *μ*_FE_ of Device #2 is slowly developed after the turn-on voltage of MoS_2_ at *V*_BG_ =  − 28.5 V which is very similar to *μ*_FE_ of Device #5. This *μ*_FE_ behavior reflects clearly that the bare MoS_2_ part in Device #2 limits the overall carrier transport in this *V*_BG_ regime. But, *μ*_FE_ rapidly increases after *V*_FB_ or *V*_CNP_, reaching *μ*_FE_ saturation (~ 100 cm^2^ V^−1^ s^−1^) at *V*_BG_ = 50 V. This mobility enhancement is mainly ascribed to Gr with low SBH in the central *n*-Gr/*n*-MoS_2_ heterojunction regime in Device #2. The high on/off current ratio of the device is further attributed to the bare MoS_2_ region in the device. As a consequence, provided that the portion of bare MoS_2_ in Device #2 shrinks as short as possible without losing the on/off current ratio, a further *μ*_FE_ improvement would expect via a device layout optimization. All devices were annealed at *T* = 150 °C for 2 h in high vacuum chamber before the electrical measurement to eliminate effect of adsorbates and interface trap sites between 2D materials and dielectrics (See Supplementary Fig. S3). Hysteresis at Gr/MoS_2_ interface was negligible from that of MoS_2_ device.

**Figure 3:**
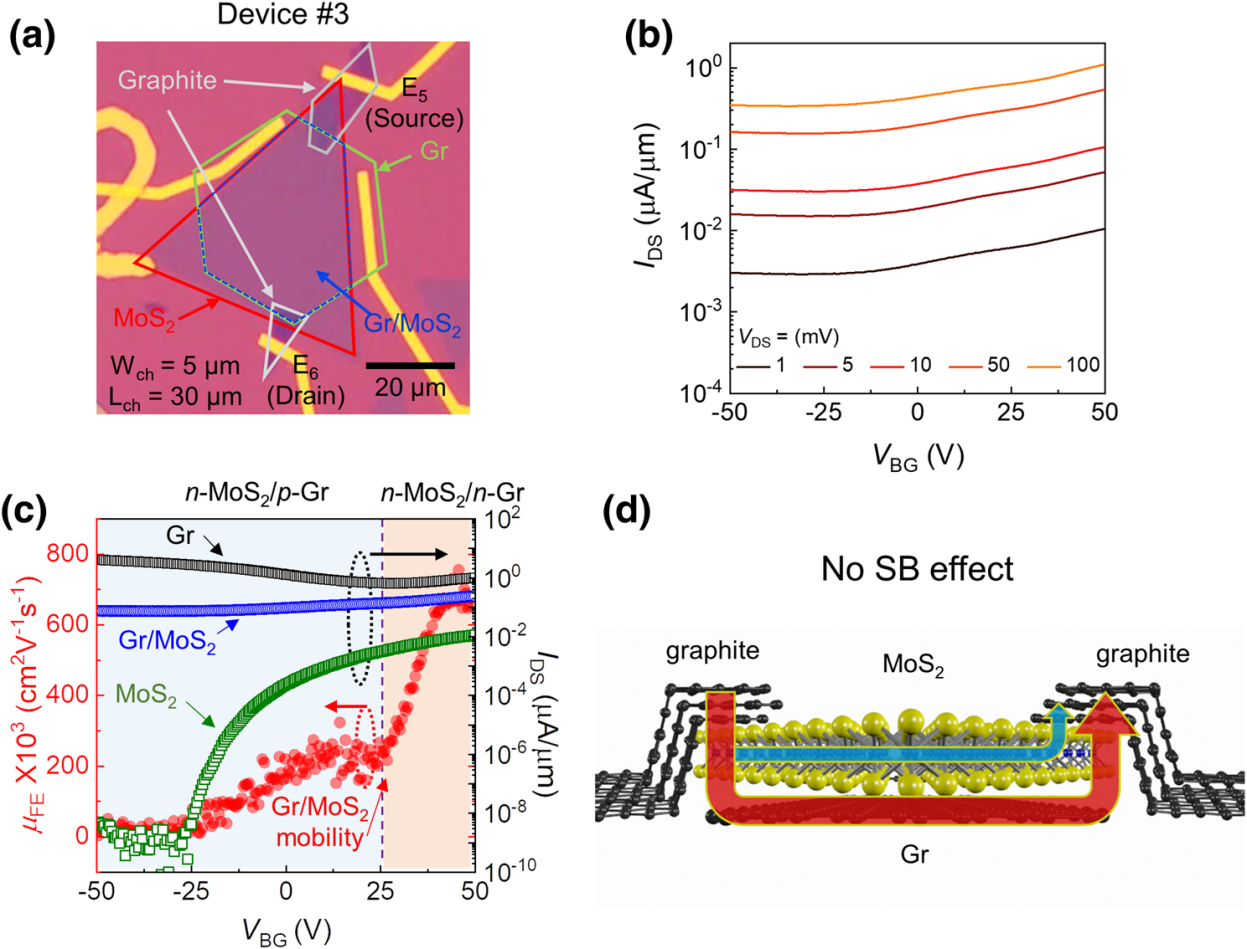
Vertical Gr/MoS_2_ heterointerface device (the bottom Gr, MoS_2_, and top graphite layers all overlapped). (**a**) Optical image of the vertical Gr/MoS_2_ heterointerface device employing graphite contact with E_5_ (source) and E_6_ (drain) (Device #3: graphite-Gr/MoS_2_-graphite, *L*/*W* = 30 μm/5 μm). (**b**) *V*_DS_-dependent transfer curve shows low on/off current ratio. (**c**) Comparison of transfer curves of the three devices (right y-axis) and *V*_BG_-dependent *μ*_FE_ (left y-axis) of Device #3 at *V*_DS_ = 0.1 V. (**d**) Current path marked by arrows for this device structure. Direct tunneling (DT) from graphite to graphene through monolayer MoS_2_ is suggested, as indicated by the red arrow. A small portion of the current flow along the MoS_2_ layer is represented by the blue arrow.

To systematically identify the transport mechanism in the Gr/MoS_2_ heterostructure, we constructed a device (Device #3: graphite-Gr/MoS_2_-graphite using metal electrodes E_5_ and E_6_) with the two graphite electrodes overlapping the lower graphene layer, intentionally excluding the bare MoS_2_ region (see Fig. 3a). It is worthy to underline that device structure of Device #3 is in contrast with that of Device #2 in particular for a spatial distance between graphite and underlying Gr (see Fig. 2e and Fig. 3d). The *V*_DS_-dependent transfer curves (Fig. 3b) were obtained from the two graphite electrodes (E_5_ and E_6_). The lack of a high on/off current ratio is ascribed to the direct tunneling (DT) current across the atomically thin monolayer MoS_2_ to the underlying graphene. The screening effect of the underlying graphene is another plausible underlying factor, resulting in weak gate modulation in the MoS_2_ channel. From the transfer curves of individual Gr, MoS_2_, and the Gr/MoS_2_ heterostructure (Fig. 3c), the charge neutrality point of Gr was found at *V*_BG_ = 27 V. General *μ*_FE_ behavior of Device #3 is very similar to that of Device #1 as shown in Fig. 2h. The high *μ*_FE_ (~ 800 cm^2^ V^−1^ s^−1^) of Device #3 in the *V*_BG_ ≥ *V*_CNP_ confirms clearly that the origin of high *μ*_FE_ is the underlying Gr. Further relevant electrical characteristics of Gr in Device #3 is discussed in Supplementary Fig. S4. In such a structure, even if the SBH at the Gr/MoS_2_ interface is modulated by *V*_BG_, the Schottky barrier effect is largely suppressed by direct tunneling at the interlayer distance, while a small portion of the total current flows along the MoS_2_ channel based on the conductivity ratio between Gr and MoS_2_ (Fig. 3d).

Now, we turn to evaluate the *V*_BG_-dependent vertical SBH in the Gr/MoS_2_ heterojunction at a given *V*_BG_ using Richardson’s equation^43^:

1 $${I}_{\mathrm{DS}}= {I}_{0}\mathrm{exp}\left(q{V}_{\mathrm{DS}}/{k}_{B}T\right)\times \left[1-\mathrm{exp}\left(-\frac{q{V}_{\mathrm{DS}}}{{k}_{B}T}\right)\right]$$

where ${I}_{0}=A{A}^{*}{T}^{3/2}\mathrm{exp}(-\frac{q{\Phi }_{\mathrm{B}}}{{k}_{B}T})$, *A* is the junction area, A* (= 54 A K^−2^ cm^−2^) is the Richardson constant^44^^,^ *n* is the ideality factor, *k*_B_ is the Boltzmann constant, *q* is the electrical unit charge, *Φ*_B_ is the effective SBH, and *T* is the absolute temperature. Rearranging Eq. 1 gives:

2 $$\mathrm{ln}\left[\frac{{I}_{\mathrm{DS}}\mathrm{ exp}\left(q{V}_{\mathrm{DS}}/{k}_{B}T\right)}{\mathrm{exp}\left(q{V}_{\mathrm{DS}}/{k}_{B}T\right)-1}\right]=\mathrm{ln}\left({I}_{0}\right)+\frac{q{V}_{\mathrm{DS}}}{n{k}_{B}T}$$

where *n* and *Φ*_B_ can be respectively determined from the slope and *y*-intercept of the $\mathrm{ln}[{I}_{\mathrm{DS}}\times \mathrm{exp}(q{V}_{\mathrm{DS}}/{k}_{\mathrm{B}}T)/(\mathrm{exp}(q{V}_{\mathrm{DS}}/{k}_{\mathrm{B}}T)-1)]$ versus the negative *V*_DS_ plot, (see Supplementary Note 2 and Fig. S5–S6 for the detailed procedures for SBH estimation).

**Figure 4:**
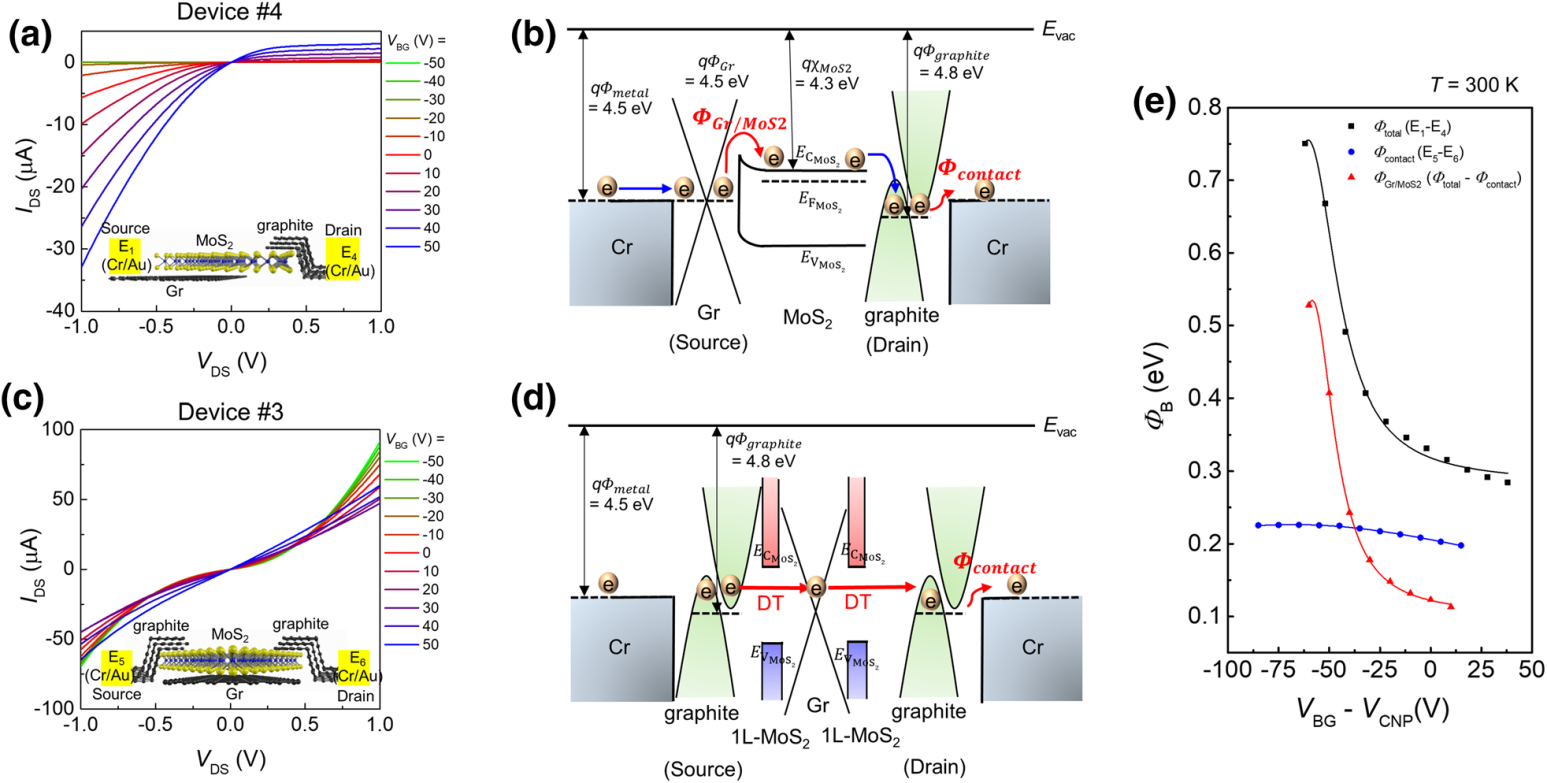
Determination of Schottky barrier height at Gr/MoS_2_ heterointerface and band diagram. (**a**) *V*_BG_-dependent output characteristic curves of Gr/MoS_2_ heterostructure device contact with E_1_ and E_4_ (Device #4: Gr-Gr/MoS_2_-graphite) and (**b**) corresponding band diagram. (**c**) *V*_BG_-dependent output characteristic curves of Device #3 and (**d**) corresponding band diagram. (**e**) Effective SBH for Gr/MoS_2_ heterointerface (*Φ*_Gr/MoS2_) as a function of *V*_BG_ − *V*_CNP_.

The *V*_BG_-dependent *I*_DS_‒*V*_DS_ curves obtained from Device #4 (Gr-Gr/MoS_2_-graphite using metal electrodes E_1_ and E_4_) are plotted in Fig. 4a. The asymmetric *I*_DS_‒*V*_DS_ curve is mainly derived from the asymmetric contact barrier (graphene contact *vs.* graphite contact). The band alignment is illustrated, along with the two dominating Schottky barriers that contribute to the total SBH (Fig. 4b). One barrier is the SB at the Gr/MoS_2_ heterojunction (${\Phi }_{\mathrm{Gr}/{\mathrm{MoS}}_{2}})$ and the other is the SB at the graphite/Cr contact junction (${\Phi }_{\mathrm{contact}})$. At the source terminal, the contact resistance at the Gr/Cr junction is negligible due to the low SB or nearly ohmic contact^42^^,^ which is consistent with the linear output characteristics illustrated in Fig. 2d. The SB was clearly formed at the Gr/MoS_2_ junction due to the difference in the work function of the two materials. Thereafter, electrons flow naturally from MoS_2_ to graphite because the energy state of the conduction band of MoS_2_ is higher (~ 4.3 eV) than that of graphite (~ 4.8 eV)^30^. This implies that the SB at the MoS_2_/graphite interface can be excluded from the total SBH estimation. Electrons are then thermally emitted to Cr by overcoming the SB at the graphite/Cr interface, where the contact barrier is formed. Therefore, the total SBH (${\Phi }_{\mathrm{total}}$) obtained from this structure is attributed to the SB at the Gr/MoS_2_ (${\Phi }_{\mathrm{Gr}/{\mathrm{MoS}}_{2}}$) heterojunction and graphite/Cr contact (${\Phi }_{\mathrm{contact}})$, where the total SBH can be expressed as$: {\Phi }_{\mathrm{total}}={{\Phi }_{\mathrm{Gr}/{\mathrm{MoS}}_{2}}+ \Phi }_{\mathrm{contact}}.$ The *I*_DS_‒*V*_DS_ curves of Device #3 present non-linear behavior in the *V*_BG_ regimes below *V*_CNP_ (‒50 V ≤ *V*_BG_ ≤ 20 V) (see Fig. 4c). The low on/off current ratio of this device (see Fig. 3b) provides clear evidence of DT, where the electrons flow through the atomically thin monolayer MoS_2_ to the underlying Gr, leading to a negligible contribution of Gr/MoS_2_ SB to the total SBH. Accordingly, ${\Phi }_{\mathrm{contact}}$ at the graphite/Cr interface could be regarded as ${\Phi }_{\mathrm{total}}$ for this device structure, where ${\Phi }_{\mathrm{total}}\simeq {\Phi }_{\mathrm{contact}}$ (see Fig. 4d). Consequently, ${\Phi }_{\mathrm{Gr}/{\mathrm{MoS}}_{2}}$ can be deduced by subtracting ${\Phi }_{\mathrm{contact}}$ from ${\Phi }_{\mathrm{total}}$. The *V*_BG_-dependent ${\Phi }_{\mathrm{total}}$, ${\Phi }_{\mathrm{contact}}$, and effective ${\Phi }_{\mathrm{Gr}/{\mathrm{MoS}}_{2}}$ at *T* = 300 K are directly compared in Fig. 4e. Increasing *V*_BG_ reduced ${\Phi }_{\mathrm{Gr}/{\mathrm{MoS}}_{2}}$ when the value of *V*_BG_ was positive in the range of 528‒116 meV, clearly indicating the large Fermi-level tunability of Gr via electrostatic gating.

## Conclusion

In conclusion, we propose that the Gr/MoS_2_ heterointerface can be employed as a high-performance electronic device. The high carrier mobility of 100 cm^2^ V^−1^ s^−1^ of Gr/MoS_2_ heterojunction device over 8–10 cm^2^ V^−1^ s^−1^ of MoS_2_ device is ascribed to the underlying Gr, which is activated when the low SBH is formed in the *n*-Gr/*n*-MoS_2_ regime. On the other hand, the high on/off current ratio of ~ 10^8^ is attributed to the bare MoS_2_ region in the heterostructure device. Furthermore, we demonstrate that the high tunability of the Fermi level of Gr allows to control the SBH at the Gr/MoS_2_ interface, resulting in distinctive carrier conduction features of the Gr/MoS_2_ heterojunction device, and confirming consequently two different conduction mechanisms.

## Methods

### Material synthesis

Monolayer Gr flakes were first synthesized on a 100 μm thick Cu foil (111) in a chemical vapor deposition (CVD) chamber under hydrogen with a low concentration of methane (0.1%, balance Ar gas) as a carbon source. The synthesized graphene with the polymer supporting layer was softly detached by bubble interception in sodium hydroxide solution and rinsed thrice with distilled water to eliminate contaminants during the chemical wet etching process. The graphene layer was then transferred onto a 300 nm-thick SiO_2_ substrate. Reactive ion etching (RIE, AFS-4RT; O_2_ plasma power of 30 mW; flow rate of 20 sccm at 10^‒3^ torr) was used to define the channel area of Gr. Monolayer MoS_2_ flakes were grown in a separate CVD chamber and transferred onto graphene strips by a water-assisted transfer method^45^. Thereafter, the exfoliated graphite flakes (~ 5 nm) were placed on the Gr/MoS_2_ heterostructure by the aligned transfer technique, in a glove box. Finally, the source/drain metal electrodes were patterned on the polymer resist via electron beam lithography, followed by metal deposition. All processes were completed within 24 h to maintain consistency in terms of the device reliability and reproducibility.

### Device fabrication

The devices were fabricated by a series of wet- and dry-cooperative transfer methods. The channel materials (single-crystal graphene and MoS_2_) were both prepared by CVD^35,46^. The graphene synthesized on copper was firstly delaminated by electrochemical bubbling and then transferred onto the target 300 nm SiO_2_/Si substrates^47^. Thereafter, the MoS_2_ grown on the SiO_2_/Si substrate was detached by chemical etching^48^. After rinsing copiously with deionized water, the MoS_2_ flakes carried by the polymethyl methacrylate (PMMA) transfer film were assembled on the dry transfer holder. The two channels were aligned in the desired positions, and MoS_2_ was brought into contact with the graphene and held at 140 ℃ for 5 min, thus isolating the PMMA film from the transfer holder. The electrical contact of MoS_2_ was improved by using graphite, which was mechanically exfoliated onto the PMMA coated 300 nm SiO_2_/Si substrates and then deposited on the designed positions inside or outside the overlapped graphene/MoS_2_ by the conventional PMMA supporting layer method^49^. Subsequently, the source and drain for the graphene channel and the contact to the graphite electrodes were patterned by e-beam lithography, followed by vapor deposition of 5/50 nm Cr/Au. For the strip graphene channel, an additional patterning step was performed by photolithography prior to O_2_-plasma dry etching.

### Optical and electrical characterization

The optical Raman spectrum of the developed Gr/MoS_2_ heterointerface device was acquired under 532 nm laser excitation with a power of 12 μW (Witec Alpha 300) in an argon-filled glove box system, where the oxygen and moisture levels were kept below 1 ppm to prevent undesired oxidation and aging of the samples. Electrical measurements were carried out using a commercial source measurement unit (Keithley 4200-SCS) under low-vacuum conditions (~ 10^−3^ torr) in a vacuum probe station, at room temperature.

## Supplementary information

Supplementary Information.
