# Supplementary material for: High-mobility junction field-effect transistor via graphene/MoS2 heterointerface
Source: Sci Rep. 2020 Aug 4;10:13101. doi: 10.1038/s41598-020-70038-6 (PMC7403303; doi:10.1038/s41598-020-70038-6)
Supplement: Supplementary file 1 — Supplementary Information. [file 41598_2020_70038_MOESM1_ESM.docx]

Supplementary Information

**High-Mobility Junction Field-Effect Transistor via Graphene/MoS_2_ Heterointerface**

Taesoo Kim^1,2,†^, Sidi Fan^1,2, †^, Sanghyub Lee^1,2^, Min-Kyu Joo^3*^ & Young Hee Lee^1,2*^

*^1^Center for Integrated Nanostructure Physics (CINAP), Institute for Basic Science (IBS), Sungkyunkwan University, Suwon 16419, Republic of Korea*

*^2^Department of Energy Science, Department of Physics, Sungkyunkwan University, Suwon 16419, Republic of Korea*

*^3^Department of Applied Physics, Sookmyung Women’s University, Seoul 04310, Republic of* Korea

***Email:** M. -K. Joo ([mkjoo@sookmyung.ac.kr](mailto:mkjoo@sookmyung.ac.kr)), Y. H. Lee ([leeyoung@skku.edu](mailto:leeyoung@skku.edu))

^†^These authors contributed equally to this work.

Note1. Field-effect carrier mobility calculation

The field-effect carrier mobility *μ*_FE_ can be calculated using the following equation^1^,

$\mu_{\mathrm{FE}}=\frac{Lg_{m}}{WC_{i}V_{\mathrm{DS}}}$ (1)

where *L* and *W* are the length and width of the channel, *g*_m_ is the transconductance extracted from *I*_DS_-*V*_BG_ transport curve, *C*_i_ is the capacitance of the gate oxide dielectric layer, and *V*_DS_ is the source-drain bias, respectively. *g*_m_ can be determined by the 1^st^ order differentiating *I*_DS_ with respect to *V*_BG_ as described in below,

$g_{m}=\frac{\partial{(I}_{DS})}{\partial{(V}_{BG})}$ (2)

where *I*_DS_ is the source-drain current and *V*_BG_ is the applied back gate bias. *C*_i_ was then finally calculated via following definition,

$C_{i}=\frac{{\varepsilon_{0}\varepsilon}_{i}}{t_{i}}$ (3)

where *ε*_0_ is the vacuum permittivity (8.854 × 10^-12^ F/m), *ε*_i_ (= 3.9)^2^ is the relative permittivity of SiO_2_, and *t*_i_ (= 300 nm) is the thickness of gate SiO_2_, respectively.

In our devices, the channel *L*/*W* ratios were obtained *via* a conventional optical microscope as 2.8 (Device #1), 3 (Device #2), 6 (Device #3), 0.48 (Device #5), and 1.86 (Device #6) using electrodes E_1_-E_2_, E_3_-E_4_, E_5_-E_6_, E_9_-E_10_, and E_7_-E_8_, respectively. Various *V*_DS_ (= 0.001, 0.005, 0.01, 0.05 and 0.1 V for Device #1 ~ #3 and #5) conditions were considered but *V*_DS_ = 1.0 V was additionally applied in the Device #5.

**Note2. Schottky barrier height calculation**

The Schottky barrier height (SBH) is calculated by rearranged Richardson equation^3^,

$$\ln\left[ \frac{I_{\mathrm{DS}} \exp\left( {qV_{\mathrm{DS}}}/{k_{B}T} \right)}{\exp\left( {qV_{\mathrm{DS}}}/{k_{B}T} \right)-1} \right]=\ln\left( I_{0} \right)+\frac{qV_{\mathrm{DS}}}{nk_{B}T} ,$$

where k_B_ is the Boltzmann constant and T is the absolute temperature. Based on the *V*_DS_-dependent $\ln{{[I}_{DS}exp(qV_{DS}}/{k_{B}T)/exp{(qV_{DS}}/{k_{B}T)-1}]}$, the *y*-intercept is simply obtained from the linear fit to the curve^4^. The results for each case are displayed in Fig. S4.


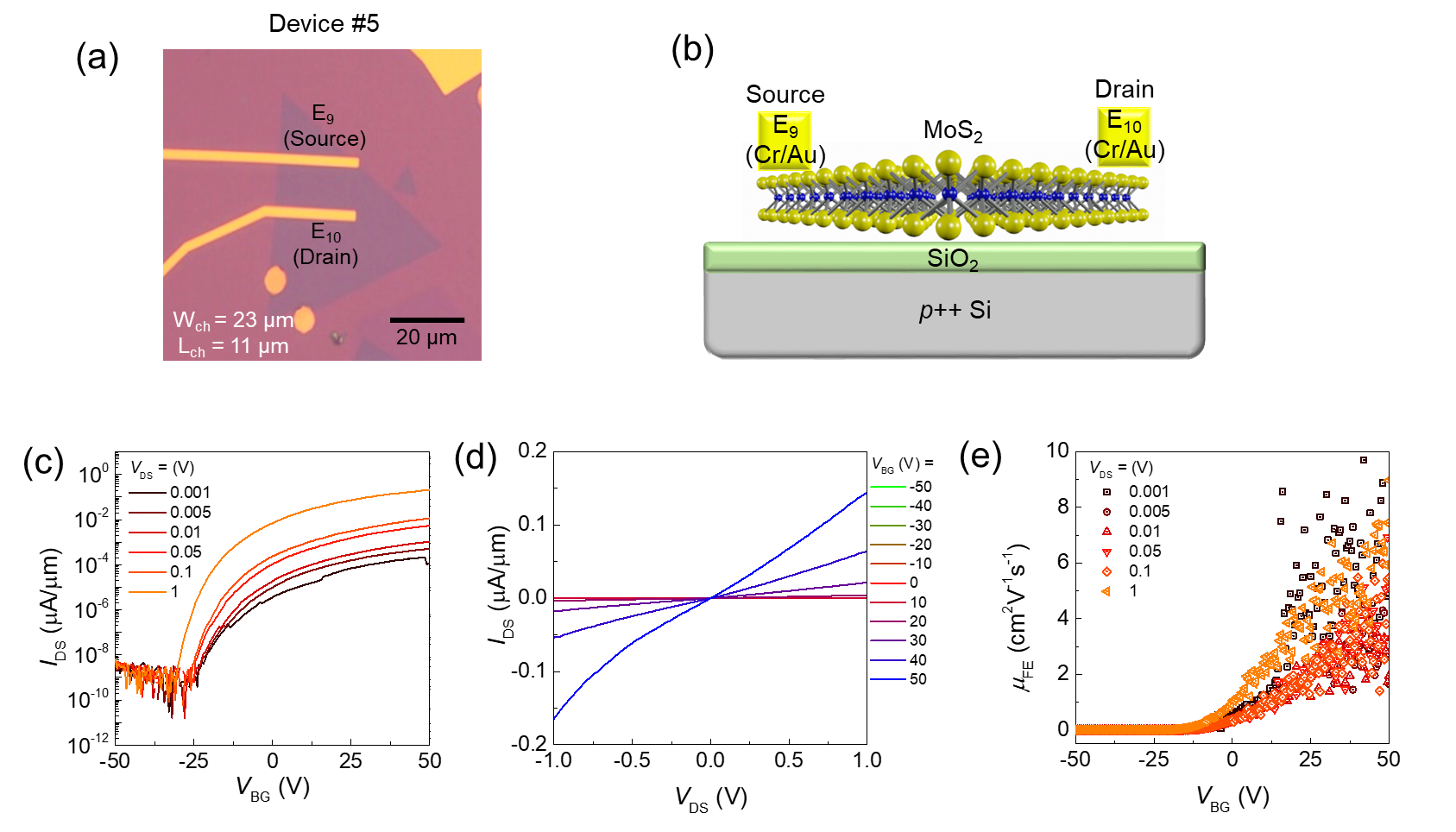


**Figure S1.** Bare MoS_2_ device (Device #5: Bare MoS_2_ device connected to E_9_ and E_10_ electrodes, *L*/*W* = 11 μm/23 μm) image and its electrical property. **(a)** Optical image of bare MoS_2_ transistor and **(b)** its conceptual image. **(c)** *V*_DS_-dependent transfer curve (*I*_DS_-*V*_BG_), **(d)** *V*_BG_-dependent output characteristic curve (*I*_DS_-*V*_DS_), and **(e)** *V*_BG_-dependent *μ*_FE_ of curves. The maximum *μ*_FE_ value is estimated ~9 cm^2^∙V^−1^∙s^−1^.


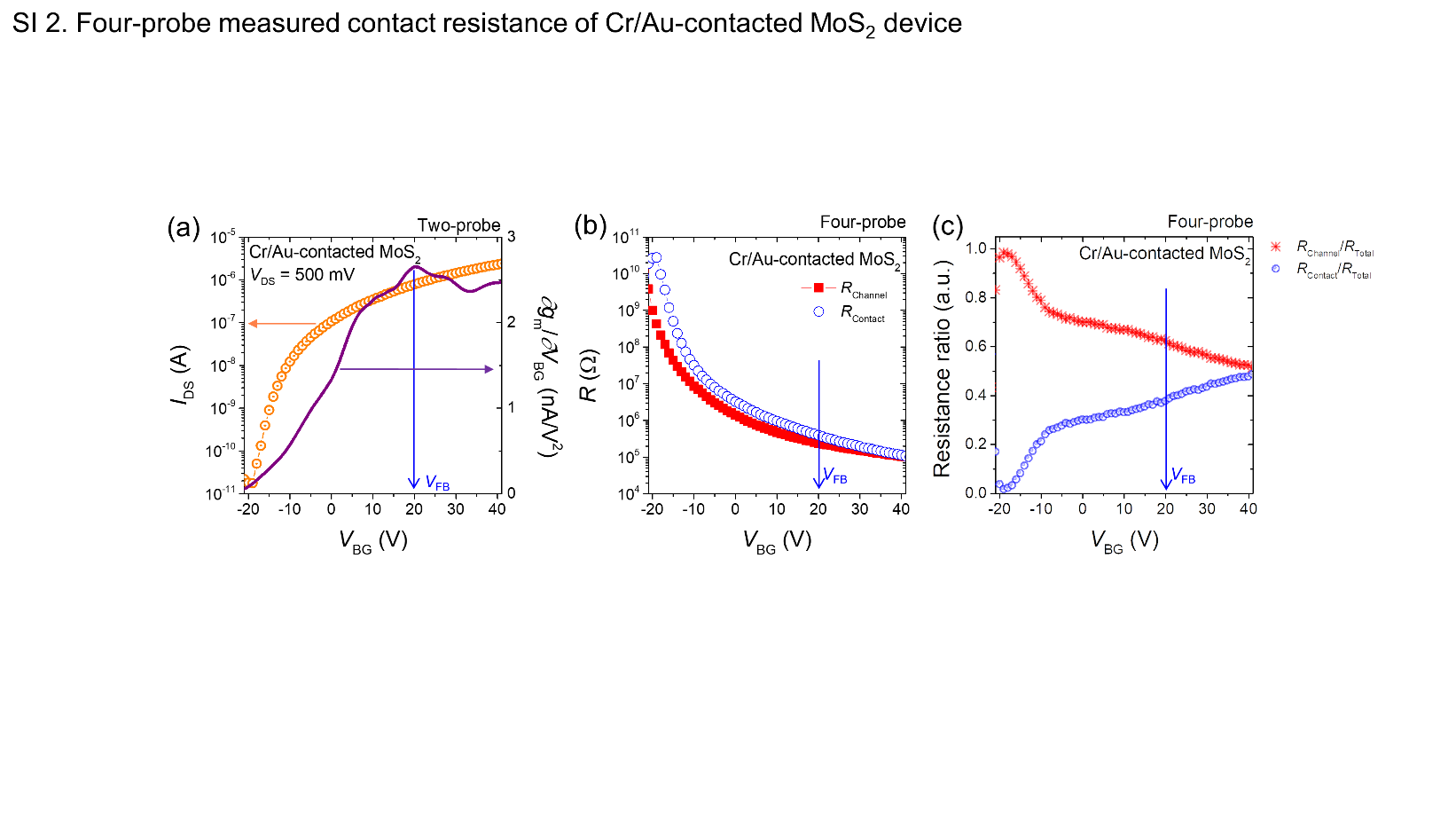


**Figure S2.** Four-probe measured contact resistance of Cr/Au-contacted MoS_2_ device. **(a)** Two-probe measured *I*_DS_-*V*_BG_ transfer curve (left y-axis) and the corresponding the second derivative of *I*_DS_ (right y-axis) of Cr/Au-contacted MoS_2_ at *V*_DS_ = 500 mV **(b)** Comparison of *V*_BG_-dependent channel (*R*_Channel_, red symbols) and contact resistance (*R*_Contact_, blue symbols). **(c)** Relative resistance ratio of *R*_Channel_/*R*_Total_ (red symbols) and *R*_Contact_/*R*_Total_ (blue symbols), where *R*_Total_ = *R*_Channel_ + *R*_Contact_.

The contact resistance (*R*_Contact_) contribution to carrier mobility (*μ*_FE_) should be excluded for a *μ*_FE_ determination. To this end, we examined the *R*_Contact_ for the Cr/Au-contacted MoS_2_ by employing the four-probe measurement (see Fig. S2a). The obtained *R*_Contact_ is ~100 kΩ (or *R*_Contact_⋅*W* ≈ 1.2 MΩ⋅μm) at *V*_BG_ = 40 V as presented in Fig. S2b. For a simple *μ*_FE_ estimation, the relative resistance ratios of *R*_Contact_/*R*_Total_ and *R*_Channel_/*R*_Total_ as a function of *V*_BG_ are presented in Fig. S2c. At *V*_BG_ larger than the flat-band voltage (*V*_FB_ = 20 V) determined from the second-derivative of *I*_DS_ methodology, the *R*_Contact_ takes almost a half of *V*_DS_ near on-current at high *V*_BG_ (see Fig. S2c), resulting in *μ*_FE_ underestimation by a factor of two. This fact implies clearly that *μ*_FE_ of the pure MoS_2_ device, i.e., Device #5, would increase to 16 ~ 20 cm^2^⋅V^−1^⋅s^−1^ after *R*_contact_ correction, but this *μ*_FE_ range is still much lower than *μ*_FE_ (~100 cm^2^⋅V^−1^⋅s^−1^) of Device #2.


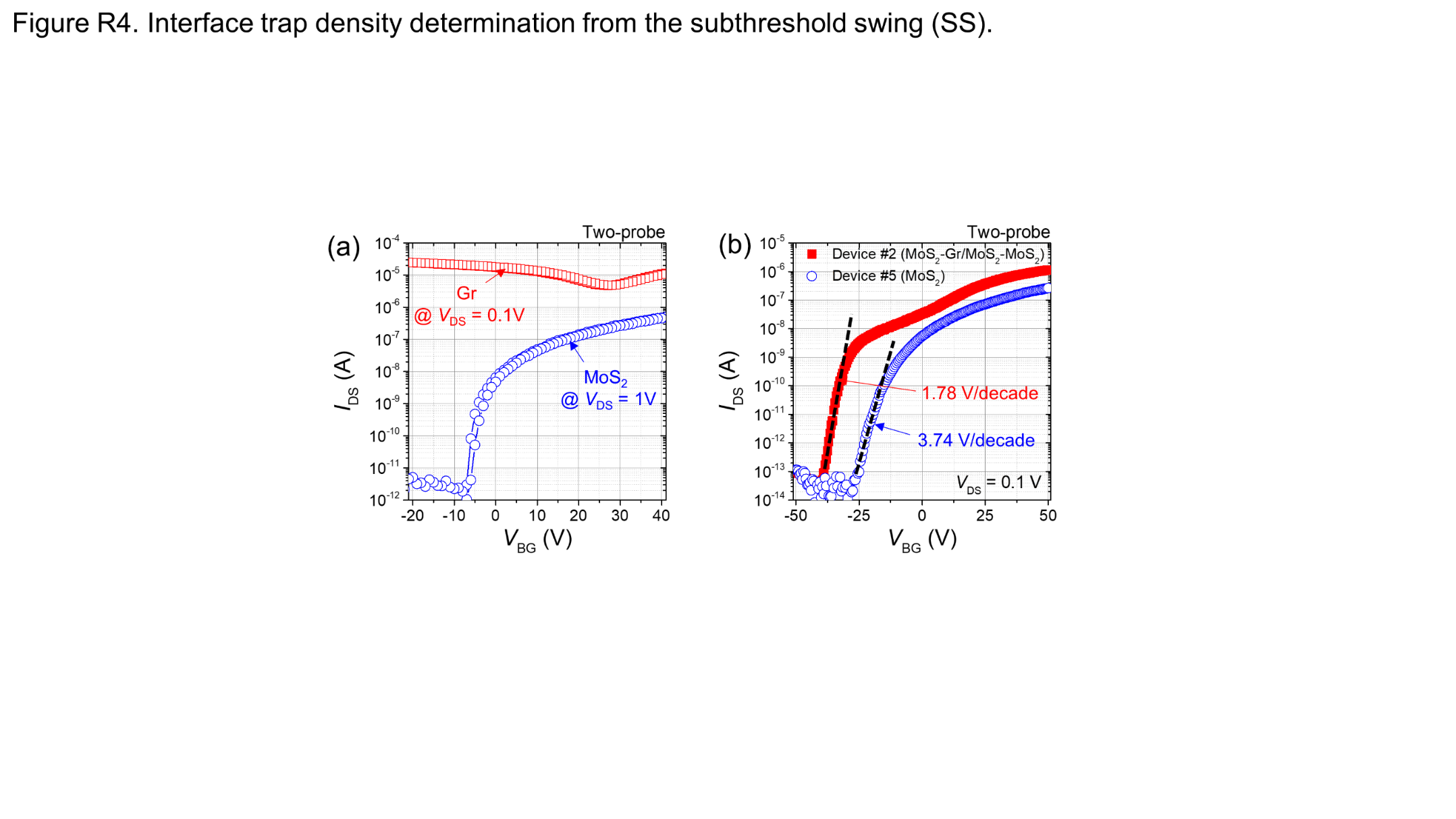


**Figure S3.** Interface trap density from the subthreshold swing (SS). (a) Generally observed double sweep *I*_DS_-*V*_BG_ measurement for Gr and MoS_2_ devices after thermal annealing at *T* = 150 ℃ for 2 hours in high vacuum chamber. (b) Two-probe measured *I*_DS_-*V*_BG_ transfer curve of Device #2 and Device #5 in this work.

Hysteresis in transfer curve *I*_DS_-*V*_BG_, originating from i) the undesired surface adsorbates in 2D materials and ii) the existence of interface trap sites between 2D materials and dielectrics, usually disappears via a proper thermal annealing process in our laboratory (see Fig. S3a). In this work, we carried out thermal annealing process at *T* = 150 ℃ for 2 hours in high vacuum chamber before the electrical measurement with a closely similar protocol and the same equipment, resulting in negligible hysteresis effects on the device under the test. We determined the interface trap density (*D*_IT_) of Device #2 (2.1 × 10^12^ cm^−2^⋅eV^−1^) and Device #5 (4.5 × 10^12^ cm^−2^⋅eV^−1^) from the subthreshold swing (*SS*) of transfer curves (see Fig. S3b), according to the following analytical model:

$$D_{IT}\approx\left( \frac{SS}{ln(10)\cdot k_{B}\cdot T}-1 \right)\frac{C_{OX}}{q},$$

where *k*_B_, *T*, *C*_OX_, and *q* denote the Boltzmann constant, absolute temperature, oxide capacitance per unit area, and electronic unit charge, respectively. The lower *D*_IT_ of Device #2 (Gr/MoS_2_ interface) than device #5 (MoS_2_ device) clearly suggests the beneficial effect of underlying graphene on *D*_IT,_. Hysteresis at Gr/MoS_2_ interface is negligible from that of MoS_2_ device.


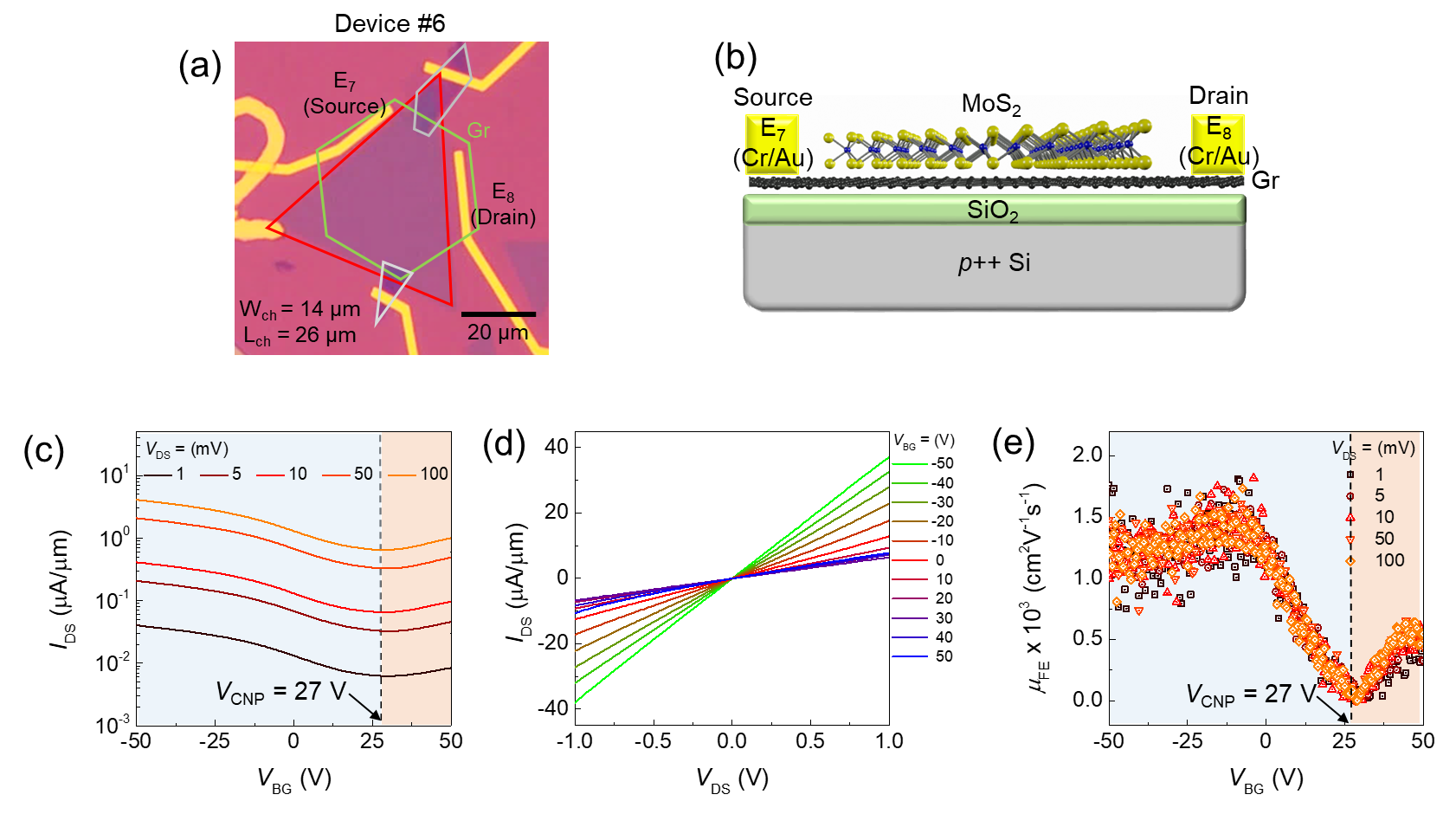


**Figure S4.** Electrical characteristics of Device #6 (Gr-Gr/MoS_2_-Gr device connected to E_7_ and E_8_ electrodes, *L*/*W* = 26 μm/14 μm). **(a)** The optical image and **(b)** its conceptual image of Device #6. **(c-d)** *I*_DS_-*V*_BG_ and *I*_DS_-*V*_DS_ characteristics. The overall electrical properties resemble the curve of the device in Fig. 2b. **(e)** The *V*_BG_-dependent *μ*_FE_ plot.


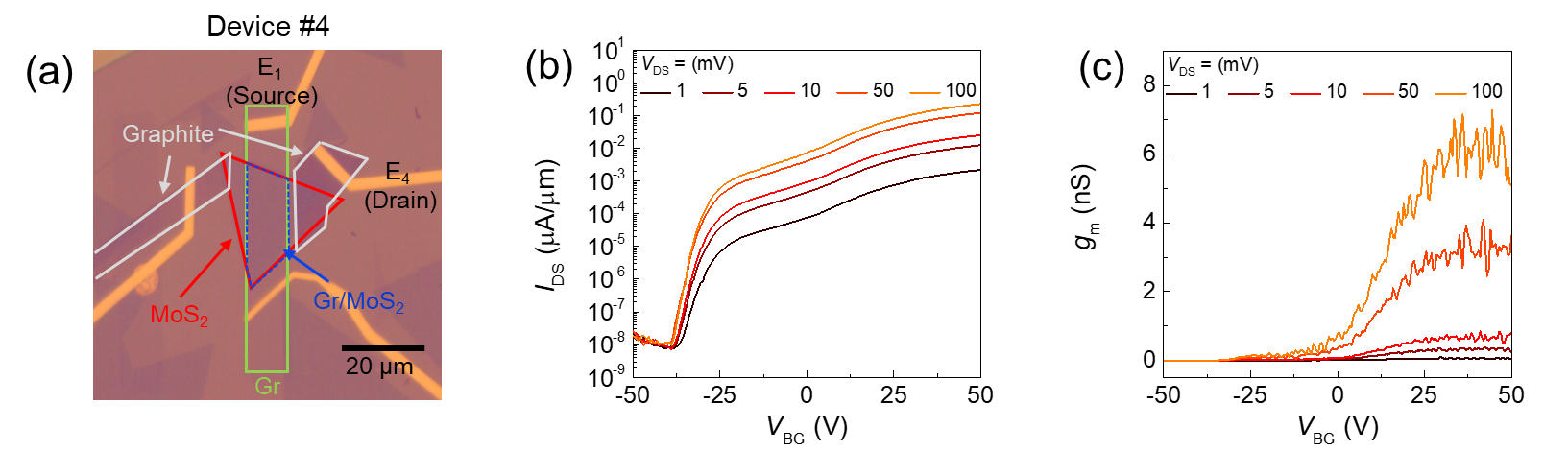


**Figure S5.** Electrical characteristics of Device #4 (Gr-Gr/MoS_2_-graphite device connected to E_1_ and E_4_ electrodes). **(a)** Optical image of Device #4 **(b)** *I*_DS_-*V*_DS_ curve and **(c)** corresponding *g*_m_.


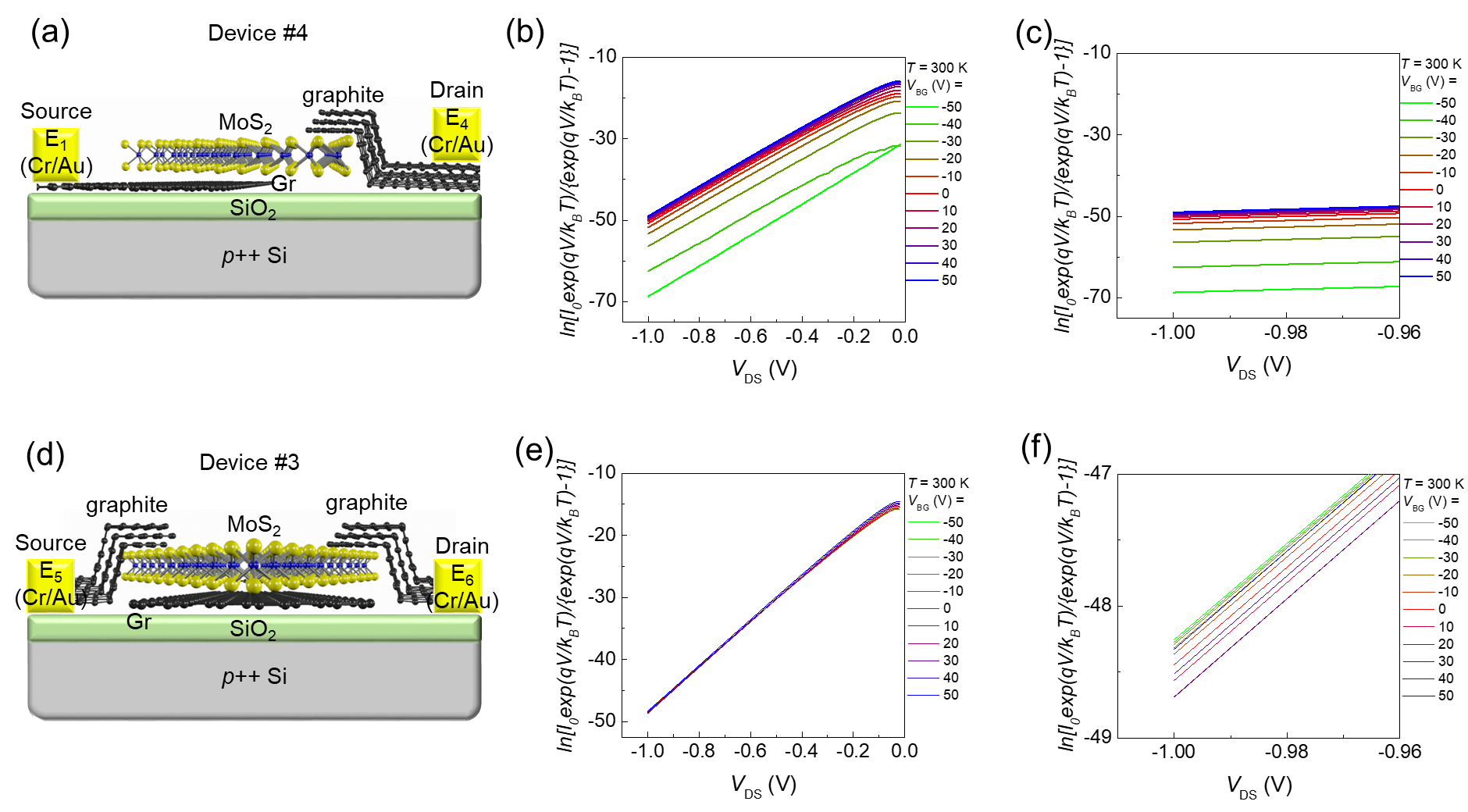


**Figure S6.** SBH calculation. **(a)** Conceptual image of Device #4 which includes both heterostructure barrier $(\Phi_{Gr/\mathrm{MoS}_{2}})$ and contact barrier ${(\Phi}_{\mathrm{contact}})$. **(b-c)** The calculated *V*_DS_-dependent$\ln{{[I}_{DS}exp(qV_{DS}}/{k_{B}T)/exp{(qV_{DS}}/{k_{B}T)-1}]}$ values and exaggerated plots in smaller *V*_DS_. **(d)** The conceptual image of Device #3 containing $\Phi_{\mathrm{contact}}$ only and (e-f) its linear fitting results.

**References**

1. Lee, Y. G. *et al*. Origin of the channel width dependent field effect mobility of graphene field effect transistors. *Microelectron.* *Eng.* **163**, 55-59, <https://doi.org/10.1016/j.mee.2016.06.004> (2016)

2. Park, S., Kim, C. H., Lee, W. J., Sung, S. & Yoon, M. H. Sol-gel metal oxide dielectrics for all-solution-processed electronics. *Mater. Sci. Eng.* *R Reports* **114**, 1-22, <https://doi.org/10.1016/j.mser.2017.01.003> (2017)

3. Wang, J. *et al*. High Mobility MoS_2_ Transistor with Low Schottky Barrier Contact by Using Atomic Thick h-BN as a Tunneling Layer. *Adv. Mater.* **28**, 8302-8308, <https://doi.org/10.1002/adma.20162757> (2016)

4. Wang, W. *et al*. Controllable Schottky barriers between MoS_2_ and permalloy. *Sci. Rep.* **4**, 1-6, <https://doi.org/10.1038/srep06928> (2014)
